# Appendicitis

**Published:** 1894-06-23

**Authors:** 


### APPENDICITIS.

Dr. Drummond draws attention[1] to various points in the anatomy and histology of the appendix. With regard to morbid anatomy there might be (1) simple adhesions; (2) enlargement of lymphoid tissue with or without cystic changes; (3) the same with pin-hole perforations, resulting probably from small follicular abscesses; (4) large perforations, either from inflammation and sloughing following pin-hole perforations, or from concretions setting up inflammation; or (5) diffuse suppurative peritonitis, either from pin-hole perforations causing abscess, which ruptured, or rapid infection of the peritoneum from the rupture caused by concretions. The clinical varieties of typhlitis corresponded to these different morbid conditions.

Hodenpyl considers[2] there are two classes of factors in the causation of acute appendicitis: (1) Predisposing factors which may vary in different cases; (2) more active factors, of which there seem to be two distinct but intimately-associated elements — (*a*) bacterial; (*b*) the less well-defined and less understood chemical factors associated with the unorganised fæcal contents. His conclusions as regards the action of bacteria are as follow: (1) The bacillus coli communis is normally present in the intestinal canal after birth, but it is never found during life outside the intestinal canal in any of the other viscera, so far as is known, without being accompanied by lesions. (2) The bacillus coli communis was found in the exudate in thirty-four out of thirty-five cases of exudative appendicitis, and in thirty-one of these it was found unassociated with any other germ. (3) The bacillus coli communis has been found as the only species of bacteria in connection with a variety of inflammatory and necrotic conditions other than appendicitis. These have almost always been associated with a demonstrable lesion of the intestinal wall, and there is evidence that under certain conditions the germ may pass through the intestinal wall without a solution of continuity. It has been abundantly shown that the germ is highly pyogenic. (4) Inoculations into animals of pure cultures of this bacillus result in inflammation, in the formation of abscesses, and sometimes produce general septic infection or intoxication.

There is one case recorded, however, in which the streptococcus pyogenes was the only organism isolated from the exudate. Of the predisposing causes of acute appendicitis stricture of the appendix is one of the most frequent. This condition, though sometimes probably the result of previous inflammatory processes, is in a large proportion of instances the result of partial retrograde evolution, the cæcal opening, which is much dilated in the infant, gradually contracting until adult age, when it is smaller than the rest of the lumen, and sometimes much constricted. The vermiform process, like other organs which undergo retrograde evolution, is very prone to become inflamed. Again, the longer the appendix the more liable it becomes to inflammatory changes. Other predisposing causes are adhesions drawing the appendix into abnormal positions, atrophy of the mucous membrane, and concretions.

[1] Lancet, Jan. 27th, 1894, p. 211. [2] New York Med. Journ., Dec. 30th, 1893; and Brit. Med. Journ., Feb. 24th, 1894.

